# Seroprevalence of hepatitis C and associated risk factors among an urban population in Haiti

**DOI:** 10.1186/1471-230X-4-31

**Published:** 2004-12-14

**Authors:** Matthew J Hepburn, Eric J Lawitz

**Affiliations:** 1 Department of Medicine, Brooke Army Medical Center, San Antonio, Texas, USA; 2 US Army Medical Research Institute of Infectious Diseases, MCMR-UIM-R (Hepburn), 1425 Porter Street, Fort Detrick, MD, 21702-5011, USA; 3 Alamo Medical Research, 621 Camden Suite 202, San Antonio, Texas, 78215, USA

## Abstract

**Background:**

The seroprevalence of hepatitis C varies substantially between countries and geographic regions. A better understanding of the seroprevalence of this disease, and the risk factors associated with seropositive status, supply data for the development of screening programs and provide insight into the transmission of the disease. The purpose of this investigation was to determine the seroprevalence of hepatitis C and associated risk factors in an urban population in Haiti.

**Methods:**

A prospective survey for hepatitis C antibodies was conducted among an urban outpatient population in Cap-Haïtien, Haiti, with a sample size of 500 subjects. An anonymous 12 question survey, with inquiries related to demographic characteristics and risk factors for HCV acquisition, was concomitantly administered with testing. These demographic and behavioral risk factors were correlated with HCV antibody status using univariate and multivariate tests.

**Results:**

The prevalence of positive HCV antibody was 22/500 (4.4%). Subjects that were anti-HCV positive had an average of 7 ± 8.6 lifetime sexual partners, compared to average of 2.5 ± 3.5 lifetime sexual partners among HCV-negative subjects (p = 0.02). In a multiple logistic regression model, intravenous drug use (OR 3.7, 1.52–9.03 95% CI) and number of sexual partners (OR 1.1, 1.04–1.20 95% CI) were independently associated with a positive HCV antibody result.

**Conclusions:**

A substantial number of subjects with HCV antibodies were detected in this population in Haiti. Further investigation into the correlation between the number of sexual partners and testing positive for hepatitis C antibodies is indicated.

## Background

The seroprevalence of hepatitis C virus (HCV) varies substantially in different geographic regions throughout the world [1]. Prior studies have suggested a low prevalence of HCV antibodies among a sample of patients in rural Haiti [2]. No cases of positive HCV antibody were detected among 485 patients in a sexually transmitted infections clinic in Jamaica [3], but 41% of hemophiliacs in Jamaica were HCV antibody positive [4]. Our hypothesis was that a higher prevalence of HCV antibodies would be detected in an urban population in Haiti.

Risk factors associated with HCV serologic status may be specific to a country or region. In particular, the role of sexual contact in the transmission of HCV appears to be influenced by characteristics and location of the population studied [5]. Therefore, examination of the risk factors associated with the presence of HCV antibodies in this population can be utilized to guide screening procedures as well as provide insight into the transmission of HCV in the context of Haitian society. A better understanding of the transmission of HCV could enhance the effectiveness of prevention efforts. This research study provides analysis of risk factors associated with hepatitis C from a population that had not been previously studied. We have observed a seroprevalence rate of 4.4% (22/500) of HCV antibodies, with intravenous drug usage and the number of sexual partners being associated with positive HCV antibodies.

## Methods

The study was approved by the Institutional Review Board at Brooke Army Medical Center (Fort Sam Houston, Texas). Subjects were recruited in the year 2000 from a healthy population utilizing hospital and clinic services in Cap-Haïtien, Haiti, which is the second largest city in the country. Subjects were recruited on presentation to the hospital laboratory for blood draws for routine laboratory tests. This laboratory was the only location in which subjects were recruited and samples obtained. Subject participation was not limited to a particular medical condition. The first 500 subjects that agreed to participate in the study were enrolled. These subjects were presenting to the hospital for services, and were not patients in a specific clinic. After informed consent was obtained, subjects completed a written 12 question survey in Creole, at the same location as where the blood was drawn. The subjects completed the survey by themselves. The questionnaire focused on demographic information and topic areas possibly associated with transmission of hepatitis C (intravenous drug use, intranasal cocaine use, blood transfusions, sexual history, number of tattoos). Intravenous drug use and cocaine use was measured on a 0–3 scale (never-rare-frequent-daily). Blood transfusions, number of sexual partners, age of first sexual intercourse and number of tattoos were quantified. Serum was obtained for testing for HCV antibody, utilizing the Abbott HCV EIA 3.0 kit™ (Abbott Laboratories, Abbott Park, Illinois). The survey information and serum results were identified only by a subject identification number, with all other identifying information removed.

Data were analyzed using univariate correlations with a Pearson's correlation coefficient. The number of sexual partners was compared between HCV-positive and negative subjects using an independent-sample t-test. Intravenous drug use between HCV-seropositive and seronegative subjects was compared using a Fisher's exact test. A multivariate logistic regression model was employed with stepwise backward elimination of non-significant variables, with HCV antibody status as the dependent variable. All of the variables from the survey were included in the model.

## Results

A total of 500 subjects were recruited and had serum tested for HCV antibody. Only two of these subjects did not complete the survey. Most subjects who were informed of the study agreed to participate, but an exact number of subjects who refused participation in the study was not determined. The background characteristics of the patient population are displayed in Table 1. Of note, few subjects (12/496) admitted intravenous drug use, and only one subject noted having a tattoo.

**Table 1:** Characteristics and survey responses of recruited subjects

**Characteristic**	**Frequency**
Sex- % males	332/498 (67%)
Age (mean ± SD in years)	33.7 ± 15.9
Years of Education (mean ± SD)	7.8 ± 5.7
Marital Status- % married	152/495 (31%)
Age of First Sexual Intercourse (mean ± SD in years)	19.1 ± 5.7
Number of Lifetime Sexual Partners (mean ± SD)	2.7 ± 4.0
Intravenous Drug Use- % with any history of usage	12/498 (2%)
Intranasal Cocaine Use- % with any history of usage	12/496 (2%)
Subjects with tattoos	1/498

The prevalence of positive HCV antibodies was 22/500 (4.4%, 95% CI 2.6–6.2%). Subjects that were anti-HCV positive had an average of 7 ± 8.6 lifetime sexual partners, compared to average of 2.5 ± 3.5 lifetime sexual partners among HCV-negative subjects (p = 0.02). There were no other statistically significant differences between the HCV antibody-positive and HCV antibody-negative groups in terms of demographic characteristics or topic areas associated with HCV infection/transmission. Among subjects with HCV antibodies, 4/22 subjects admitted intravenous drug use compared to 8/476 among HCV-antibody negative subjects (p < 0.001). A similar result was observed for rates of intranasal cocaine use among HCV-antibody positive (4/22) vs. HCV-antibody negative (8/474) subjects (p < 0.001). Of the 22 subjects with positive HCV antibodies, 12 subjects denied intravenous drug use, had no tattoos, and had either 1 or 2 lifetime sexual partners. Five subjects with positive HCV antibodies denied intravenous drug use and had >10 lifetime sexual partners.

Intravenous drug use (r = 0.26, p < 0.001), intranasal cocaine use (r = 0.29, p < 0.001) and the number of lifetime sexual partners (r = 0.24, p < 0.001) were the only three variables with a statistically significant correlation with the presence of HCV antibodies on univariate analysis. The number of lifetime sexual partners had some correlation with intravenous drug use (r = 0.12, p = 0.009) and intranasal cocaine use (r = 0.13, p = 0.004). There was a very close correlation between intravenous drug use and intranasal cocaine use (r = 0.99, p < 0.001). Therefore, intranasal cocaine use was not incorporated into the multivariate model due to concerns about co-linearity. In the multiple logistic regression model with backwards elimination, intravenous drug use (OR 3.7, 1.52–9.03 95% CI) and number of sexual partners (OR 1.1, 1.04–1.20 95% CI) were independently associated with a positive HCV antibody result.

## Discussion

We observed a prevalence of 4.4% of HCV antibodies in an urban population in Haiti. Additionally, we found that HCV antibodies were associated with intravenous drug use as would be expected, but also with increasing number of lifetime sexual partners. It is conceivable that subjects who reported higher number of sexual partners were more likely not to admit past intravenous drug usage. However, it is also possible that this finding represents sexual transmission of hepatitis C among subjects with multiple prior sexual partners. Finally, we observed HCV antibodies were present is some subjects who denied intravenous drug use and had less than 3 lifetime sexual partners.

A recent survey of the influences on HIV preventive behaviors among youth in Haiti observed that 80% of males and 42% of females self-disclosed sexual activity [6]. This survey noted a mean age of first intercourse of 13.1 years, a number lower than our observations (19.1 years). This study also noted that condom use was infrequent in the surveyed population (18% of subjects reported always or sometimes using a condom), while 43% reported 3 or more lifetime sexual partners. The applicability of these data to our study population is limited because the age difference between this population and the population of our study. Although the results of this investigation do not directly describe the population of our study, we might extrapolate that our population would be unlikely to have a high rate of condom usage.

Only a single prior study in Haiti suggested that HCV antibodies were rare [2]. The most obvious difference between our results and this prior study was that the previous study was performed on rural subjects, while our data was collected in an urban setting. It is possible that intravenous drug abuse would be more prevalent in an urban environment, which could partially explain the observed differences in seroprevalence. The previous study was also conducted more than 10 years before our research.

Intravenous drug use does not exclusively explain the prevalence of HCV antibodies in the studied population. Although intravenous drug use was associated with an increased likelihood of having HCV antibodies (as has been thoroughly documented [1]), only four of the 22 subjects with HCV antibodies admitted drug use. Additionally, none of the HCV positive subjects had tattoos. These findings suggest either that self-reported drug use underestimates the prevalence of drug use in the population, or another mode of transmission of HCV has occurred.

The degree to which sexual transmission of HCV occurs is exceedingly controversial [5,7]. Studies in monogamous relationships suggest that sexual transmission of hepatitis C occurs very rarely [8]. Seroprevalence studies from sexually-transmitted diseases clinics describe a variable amount of HCV positive subjects, which tends to be low when injection drug users are excluded [9,10]. The number of sexual partners has been previously associated with increasing risk of HCV exposure [11]. We also observed the association between increasing number of sexual partners and the likelihood of having HCV antibodies. This association was observed even when controlling for other variables in a multivariate model. Our data suggest that sexual transmission of hepatitis C may occur more frequently in persons with multiple sexual partners. However, additional larger studies directed at evaluating HCV-infected persons with multiple sexual partners are needed. All studies on the sexual transmission of hepatitis C (including our study) are limited by the potential of the confounding variable of shared toothbrushes, razor blades and other items among sexual partners.

Our findings are limited by the lack of information regarding active HCV infection. The presence of HCV antibodies only indicates prior exposure, and definitive documentation of active HCV infection requires detection of virus in the bloodstream utilizing HCV RNA polymerase chain reaction testing. However, since most patients exposed to hepatitis C develop chronic infection [12], HCV antibody testing provides a reasonable estimate of the amount of HCV infection in a population. Other limitations of the study are linked to the difficulties inherent in self-reporting of behaviors such as sexual activity and drug use. Additionally, we were unable to obtain information on subjects that refused participation in the study, which may limit the representativeness of this population. It is also possible that the patients receiving blood draws in the Cap-Haïtien health clinic may not be representative of the population of Haiti, or even the urban population of Haiti. Finally, there are other possible alternative sources of percutaneous exposure to HCV, such as medical injections by alternative practitioners or other medical, surgical or dental procedures.

In conclusion, we provide seroprevalence data of HCV antibodies in an urban population in Haiti. These data are useful for understanding the risks of transfusion in Haiti if the blood has not been previously screened for HCV. This information contributes to our understanding of the worldwide prevalence of hepatitis C, which allows for informed decisions regarding the priorities of funding for the treatment and prevention of this infection. Additionally, we observed the number of sexual partners may be related to a greater likelihood of having HCV antibodies, but some subjects who denied intravenous drug use and had few lifetime sexual partners still had HCV antibodies. These findings could be utilized to foster consideration of new studies into some of the risk factors that are not clearly understood (such as procedures by medical, dental or alternate practitioners). These results suggest that further study into the mode of transmission of hepatitis C should focus on patients with a high number of lifetime sexual partners but no evidence of intravenous drug use.

## Competing interests

Matthew J. Hepburn, MD: no competing interests to declare.

Eric J. Lawitz, MD: Dr. Lawitz has received research grants to conduct investigator-initiated research from Schering-Plough Corporation (Kenilworth, New Jersey).

## Author's contributions

MH was involved in study design, data analysis, and manuscript preparation. EL was involved in study design, data collection, data analysis, and manuscript preparation.

## Pre-publication history

The pre-publication history for this paper can be accessed here:


